# Controlling coherence via tuning of the population imbalance in a bipartite optical lattice

**DOI:** 10.1038/ncomms6735

**Published:** 2014-12-11

**Authors:** M. Di Liberto, T. Comparin, T. Kock, M. Ölschläger, A. Hemmerich, C. Morais Smith

**Affiliations:** 1 Institute for Theoretical Physics, Centre for Extreme Matter and Emergent Phenomena, Utrecht University, Leuvenlaan 4, 3584CE Utrecht, The Netherlands; 2 Laboratoire de Physique Statistique, École Normale Supérieure, UPMC, Université Paris Diderot, CNRS, 24 rue Lhomond, 75005 Paris, France; 3 Institut für Laser-Physik, Fachbereich Physik, Universität Hamburg, Luruper Chaussee 149, 22761 Hamburg, Germany

## Abstract

The control of transport properties is a key tool at the basis of many technologically relevant effects in condensed matter. The clean and precisely controlled environment of ultracold atoms in optical lattices allows one to prepare simplified but instructive models, which can help to better understand the underlying physical mechanisms. Here we show that by tuning a structural deformation of the unit cell in a bipartite optical lattice, one can induce a phase transition from a superfluid into various Mott insulating phases forming a shell structure in the superimposed harmonic trap. The Mott shells are identified via characteristic features in the visibility of Bragg maxima in momentum spectra. The experimental findings are explained by Gutzwiller mean-field and quantum Monte Carlo calculations. Our system bears similarities with the loss of coherence in cuprate superconductors, known to be associated with the doping-induced buckling of the oxygen octahedra surrounding the copper sites.

## Abstract (web summary)

Ultracold atoms in optical lattices are a versatile platform for modelling simplified physical systems. By tuning structural deformations in bipartite optical lattices, Di Liberto *et al*. induce superfluid-to-Mott-insulator phase transitions that may shed light on condensed-matter systems such as the cuprates.

## Graphical abstract

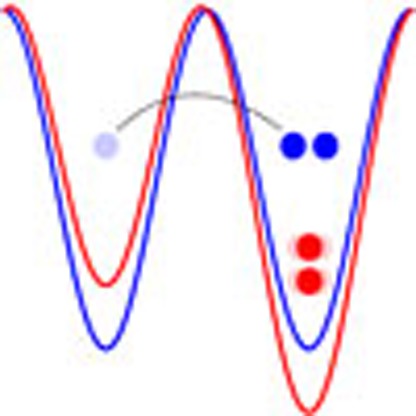

Rapid and precise control of transport properties are at the heart of many intriguing and technologically relevant effects in condensed matter. Small changes in some external parameters, for example, an electric or a magnetic field, may be used to significantly alter the mobility of electrons. Prominent examples are field effect transistors^1^ and systems showing colossal magneto-resistance^2^. Often, the control is achieved via structural changes of the unit cell, leading to an opening of a band gap. In iron-based superconductors, the variation of pressure is a well-known technique to control their transport properties^3^. In certain high-*T*_c_ superconductors, pulses of infrared radiation, which excite a mechanical vibration of the unit cell, can for short periods of time switch these systems into the superconducting state at temperatures at which they are actually insulators^4^. In La-based high-*T*_c_ cuprates, the drastic reduction of *T*_c_ at the doping value of *x*=1/8, known as ‘the 1/8 mystery’, is connected to a structural transition that changes the lattice unit cell^5^.

Ultracold atoms in optical lattices provide a particularly clean and well-controlled experimental platform for exploring many-body lattice physics^6^. Schemes for efficient manipulation of transport properties can be readily implemented and studied with great precision. In conventional optical lattices, tuning between a superfluid and a Mott insulating phase has been achieved by varying the overall lattice depth *V*_0_, with the consequence of changing the height of the tunnelling barriers and the on-site contact interaction energy^7^. The equivalent is not easily possible in condensed-matter systems, since the lattice depth is practically fixed.

In this work, we present an ultracold atom paradigm, where tuning the system between a superfluid and a Mott insulator becomes possible via controlled distortion of the unit cell. This distortion acts to adjust the relative depth Δ*V* between two classes of sites (denoted by A and B) forming the unit cell and allows us to drive a superfluid-to-Mott insulator transition without altering the average lattice depth. We can access a rich variety of Mott insulating states with different integer populations of the A and B sites, which give rise to a shell structure in the finite harmonic trap potential, leading to characteristic features in the visibility of Bragg maxima in momentum spectra. We compare our observations with quantum Monte Carlo (QMC) and Gutzwiller mean-field calculations, thus obtaining a detailed quantitative understanding of the system. In the following, we first describe our experimental set-up; then, we theoretically investigate the behaviour of the visibility for two different cases: first, for fixed barrier height *V*_0_, by varying Δ*V* (bipartite lattice), and second, for Δ*V*=0 (monopartite lattice), by tuning the lattice depth *V*_0_. Although monopartite lattices have been previously studied in great detail, and QMC calculations have provided a good fitting of the visibility curve measured experimentally^8^, here we show more accurate data and argue that the main features of the curve can be understood in terms of a precise determination of the onset of new Mott lobes in the phase diagram.

## Results

### Description of the experimental set-up

**Figure 1:**
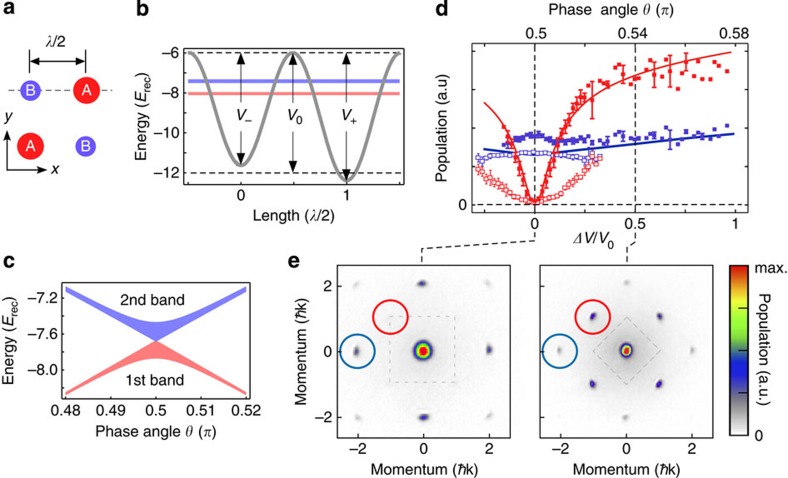
Lattice potential. (**a**) Sketch of the lattice geometry within the *xy*-plane. *λ*=1,064 nm denotes the wavelength of the laser light. (**b**) The potential along the dashed trajectory in **a** is plotted for *θ*=0.51*π* and *V*_0_=6*E*_rec_ (thick grey line) with the first and second bands represented, respectively, by the red and blue horizontal bars. (**c**) The first two bands are plotted versus *θ* for *V*_0_=6*E*_rec_. (**d**) The red and blue squares show the relative number of atoms (normalized to the total particle number and plotted versus Δ*V*/*V*_0_) associated with the Bragg peaks enclosed by red and blue circles in **e**, respectively. The filled (open) squares are recorded for *V*_z,0_=0 (*V*_z,0_=22*E*_rec_). The error bars indicate the statistical errors for five measurements. The solid lines are determined by a full-band calculation (neglecting interaction) with no adjustable parameters. (**e**) Momentum spectra (*V*_0_=6*E*_rec_, *V*_z,0_=0) are shown with Δ*V*=0 (left) and Δ*V*/*V*_0_=0.5 (right) with the respective first Brillouin zones imprinted as dashed rectangles.

We prepare an optical lattice of ^87^Rb atoms using an interferometric lattice set-up^9^,^10^,^11^,^12^. A two-dimensional (2D) optical potential is produced, comprising deep and shallow wells (A and B in Fig. 1a) arranged as the black and white fields of a chequerboard. In the *xy*-plane, the optical potential is given by *V* (*x, y*)=−*V*_0_ [cos^2^(*kx*)+cos^2^(*ky*)+2cos(*θ*) cos(*kx*) cos(*ky*)], with the tunable well depth parameter *V*_0_ and the lattice distortion angle *θ*. An additional lattice potential *V*_z_ (*z*)≡−*V*_z,0_ cos^2^(*kz*) is applied along the *z*-direction. To study an effectively 2D scenario, *V*_z,0_ is adjusted to 29*E*_rec_, such that the motion in the *z*-direction is frozen out. Here, *k*≡2*π*/*λ*, *E*_rec_≡*ħ*^2^*k*^2^/2*m*, *m* denotes the atomic mass and *λ*=1,064 nm is the wavelength of the lattice beams. Apart from the lattice, the atoms experience a nearly isotropic harmonic trap potential. Adjustment of *θ* permits controlled tuning of the effective well depths of the deep and shallow wells *V*_±_≡*V*_0_(1±cos(*θ*))^2^ and their difference Δ*V*≡*V*_+_−*V*_−_=4*V*_0_cos(*θ*) (see Fig. 1b). The effective mean well depth 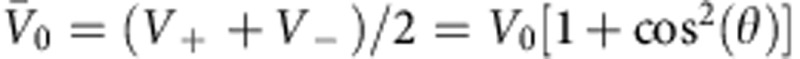 is only weakly dependent on *θ*. For example, within the interval 0.46<*θ*/*π*<0.54 one has cos^2^(*θ*)<0.015 and hence 
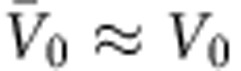
. Tuning of *θ* significantly affects the effective bandwidth, as shown in Fig. 1c. At *θ*=*π*/2, the A- and B wells become equal, which facilitates tunnelling as compared with values *θ*≠*π*/2, where the broad lowest band of the *θ*=*π*/2-lattice splits into two more narrow bands.

We record momentum spectra, which comprise pronounced Bragg maxima with a visibility 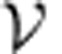 (specified in the Methods section) depending on the parameters *V*_0_ and Δ*V*. The distribution of Bragg peaks reflects the shape of the underlying first Brillouin zone, which changes size and orientation as Δ*V* is detuned from zero. This is illustrated in Fig. 1d,e. In Fig. 1e two spectra recorded for Δ*V*=0 (left) and Δ*V*/*V*_0_=0.5 (right) are shown. For Δ*V*=0 (the special case of a monopartite square lattice), the increased size of the first Brillouin zone gives rise to destructive interference, such that the ±(1, ±1)*ħk*-Bragg peaks indicated by the red circle vanish. As Δ*V* is detuned from zero, a corresponding imbalance of the A- and B populations yields a retrieval of the ±(1, ±1)*ħk*-Bragg peaks. This is shown in Fig. 1d for the case of approximately vanishing interaction energy per particle *U*≈0 (*V*_z,0_=0) by the filled red squares and for *U*≈0.3*E*_rec_ (*V*_z,0_=22*E*_rec_) by the open red squares. It is seen that the interaction energy significantly suppresses the formation of a population imbalance and corresponding ±(1, ±1)*ħk*-Bragg peaks.

### Model

For low temperatures and for large lattice depths *V*_0_, the system is described by the inhomogeneous Bose–Hubbard model^13^,^14^


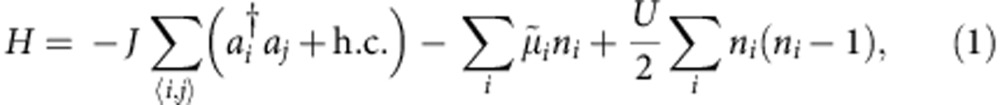


where *J* is the coefficient describing hopping between nearest-neighbour sites, *U* accounts for the on-site repulsion and 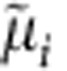 is a local chemical potential, which depends on the frequency 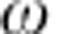 of the trap and on the sublattice: 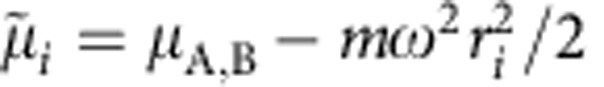. The ratio *U*/*J* is a monotonously increasing function of *V*_0_/*E*_rec_.

### Bipartite lattice Δ*V*≠0

**Figure 2:**
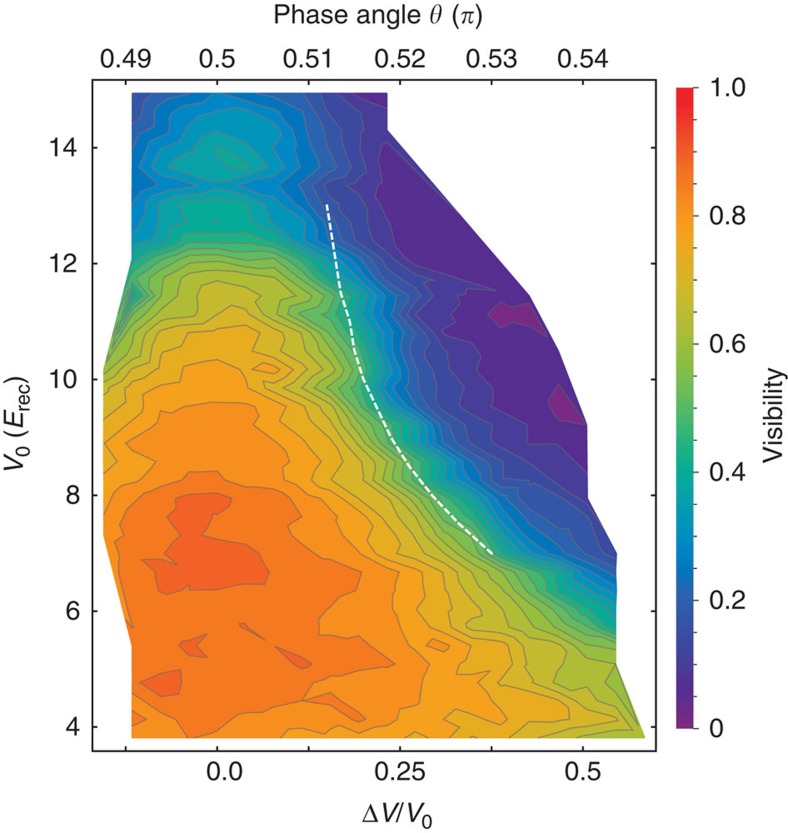
Visibility measurements in the bipartite lattice. The visibility (parametrized by the colour code shown on the right edge) is plotted as a function of the well depth parameter *V*_0_ (measured in units of the recoil energy *E*_rec_) and the potential energy offset difference Δ*V* between shallow and deep wells in the bipartite lattice. The dashed line corresponds to the theoretical calculation of the points where the fraction of particles *n*_B_=∑_*i*ε*B*_*n*_*i*_/*N* of the B sublattice vanishes (*n*_B_<5.5 × 10^−3^). The grid with the experimental points is shown in Supplementary Fig. 9.

The visibility measured for fixed *V*_0_ as a function of Δ*V* (see Fig. 2) exhibits a region of rapid decrease. When the lattice barrier is large, for example, *V*_0_=12*E*_rec_, a modest detuning Δ*V*~0.25*V*_0_ is able to completely destroy phase coherence with the consequence of a vanishing visibility. At smaller barrier heights, for example, *V*_0_=6*E*_rec_, superfluidity remains robust up to significantly larger values of Δ*V*. To explain this behaviour, we performed a mean-field calculation using the Gutzwiller technique^15^ for the Bose–Hubbard model given by equation (1). The values of *J* and Δ*μ*=*μ*_A_−*μ*_B_ have been estimated from the exact band structure and *U* has been calculated within the harmonic approximation. The total number of particles has been fixed to *N*=2 × 10^3^ and the trap frequency takes into account the waist of the laser beam (see Methods and Supplementary Note 1). We performed large-scale Gutzwiller calculations in presence of a trap, thus going beyond local density approximation^16^,^17^ (see Methods).

**Figure 3:**
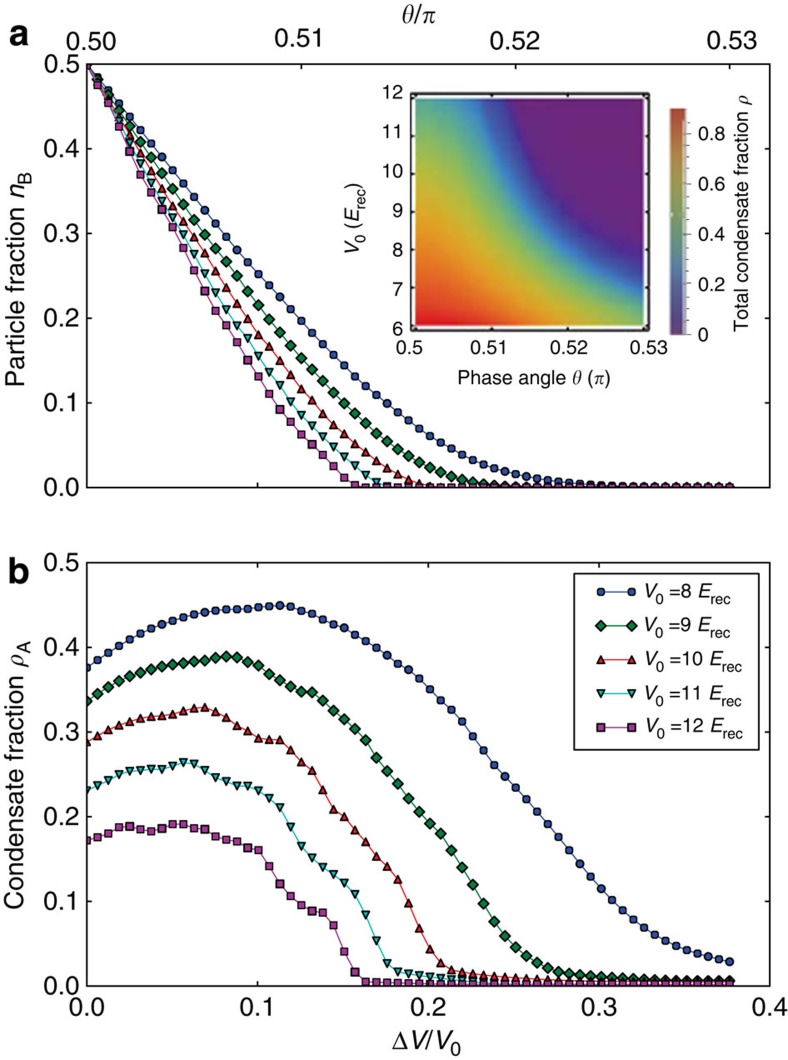
Gutzwiller results in the trap. (**a**) Particle number fraction on the B sites (*n*_B_). The inset shows the total condensed fraction *ρ*=∑_*i*_*ρ*_*i*_/*N*. (**b**) Condensate fraction on the A sites (*ρ*_A_=∑_*i*ε*A*_*ρ*_*i*_/*N*, where 
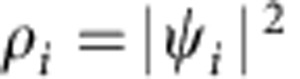
, with 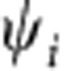 the mean-field order parameter) as a function of Δ*V* for increasing values of *V*_0_ and fixed total number of particles *N*=2 × 10^3^, calculated with the Gutzwiller ansatz. The key shows the colour code for both, the curves in **a** and **b**.

In Fig. 3a, we show the evolution of the fraction of particles in the B sites (which we assumed to be the shallow wells). As Δ*V* increases, the number of bosons in the B sites decreases because of the excess potential energy required for their population. Within the tight-binding description, this is captured by the increased chemical potential difference between A- and B sites as Δ*V* grows. Our calculations predict a critical value Δ*V*_c_ for which the population of the B sublattice vanishes. As shown in Fig. 3a, Δ*V*_c_ becomes smaller as *V*_0_ increases. This corresponds to the observation in the phase diagram shown in Supplementary Fig. 5 and discussed in Supplementary Notes 2 and 3 that the area covered by the Mott insulating regions with vanishing B populations (filling *g*_B_=0) increases as the hopping amplitude is reduced. The critical values Δ*V*_c_ for different values of *V*_0_ are also shown in Fig. 2 as a dashed white line on top of the experimental data for the visibility. This line consistently lies on experimental points corresponding to constant visibility (
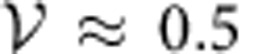
), where phase coherence is rapidly lost, and suggests the onset of a new regime.

In Fig. 3b it is shown that, in addition to the population of the B sites, also the condensate fraction at the A sites approaches zero beyond the critical value Δ*V*_c_ (see the inset in Fig. 3a for the total condensed fraction); in this regime, the density profile displays only sharp concentric Mott shells of the form (*g*_A_, *g*_B_)=(*g*, 0) where the integer filling *g* of the Mott regions can reach *g*=4 (see Supplementary Fig. 6). This can be understood by considering that in the new regime where B sites are empty, the particles populating A sites can only delocalize (and thus establish phase coherence) by hopping through the intermediate B sites. Since these are second-order processes, they are highly suppressed when Δ*μ* is large enough, and the system has to become an imbalanced Mott insulator.

**Figure 4:**
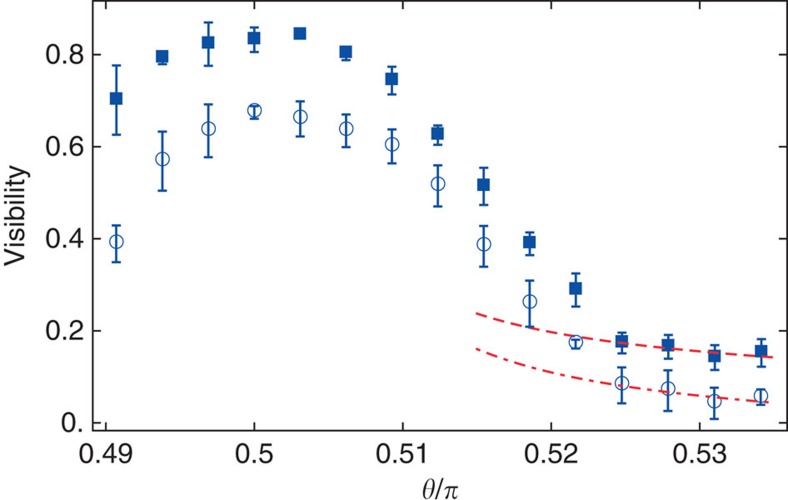
Comparison of the measured visibility with the theory at large imbalance. The data shown are for *V*_0_=10.8 *E*_rec_ (squares) and *V*_0_=11.44 *E*_rec_ (circles). The red dashed (dash-dotted) line is obtained by fitting the last four data points with equation (3) using the average filling 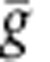 as a fitting parameter. We obtain respectively 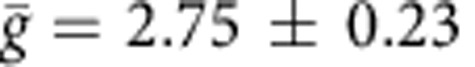 and 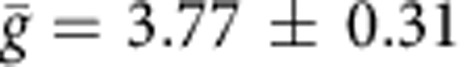. The data for *V*_0_=10.8 *E*_rec_ are shifted along the vertical axis by 0.1. The error bars represent the statistical variance of typically 4–5 independent measurements.

In the new Mott insulating regime, particle–hole pairs are responsible for a non-vanishing visibility, as in the conventional case in absence of imbalance^18^. By performing the perturbation theory on top of the ideal Mott insulating state |*MI*›=Π_*i*εA_|*g*›_*i*_Π_*j*εB_|0›_*j*_, the ground state can therefore be written as (see Supplementary Note 4).


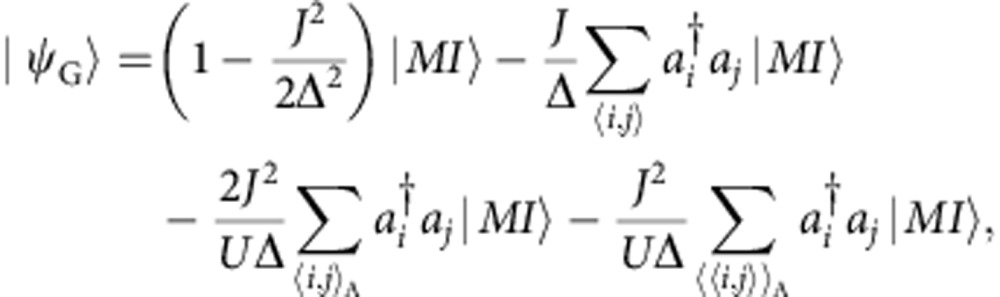


where Δ≡*U*(*g*−1)+Δ*μ*. The first term is simply the unperturbed term with a wavefunction renormalization, whereas the linear term in *J* describes particle–hole pairs with the particle sitting on the A site and the hole in the neighbour B site, or vice versa. The last two terms are second-order processes that involve intermediate B sites and describe particle–hole pairs within the A sublattice only. This ground state leads to the visibility


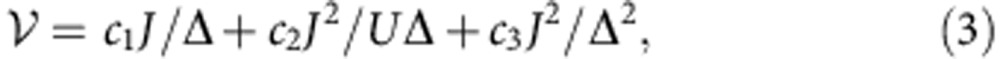


where 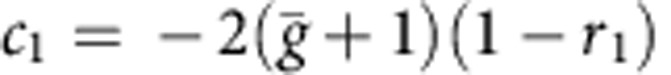, 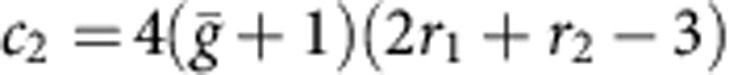, 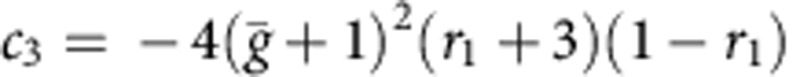, with 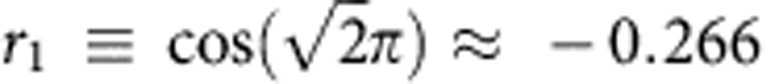 and 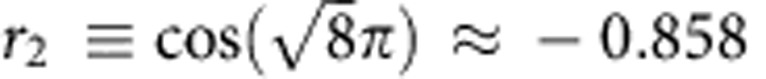. By using the average filling 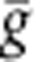 in the trap as a fitting parameter, we found that the theoretical visibility curve compares reasonably well with the experimental data both in magnitude and scaling behaviour, with an average filling of the order 
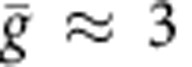
 (see Fig. 4). A perturbative description of the visibility data for large *θ* by means of equation (3) is only possible in a window *V*_0_≈11±1 *E*_rec_, where sufficient data points are available in the low-visibility tail with values of the visibility large enough to be measured with sufficient precision to allow fitting.

### Monopartite lattice Δ*V*=0

**Figure 5:**
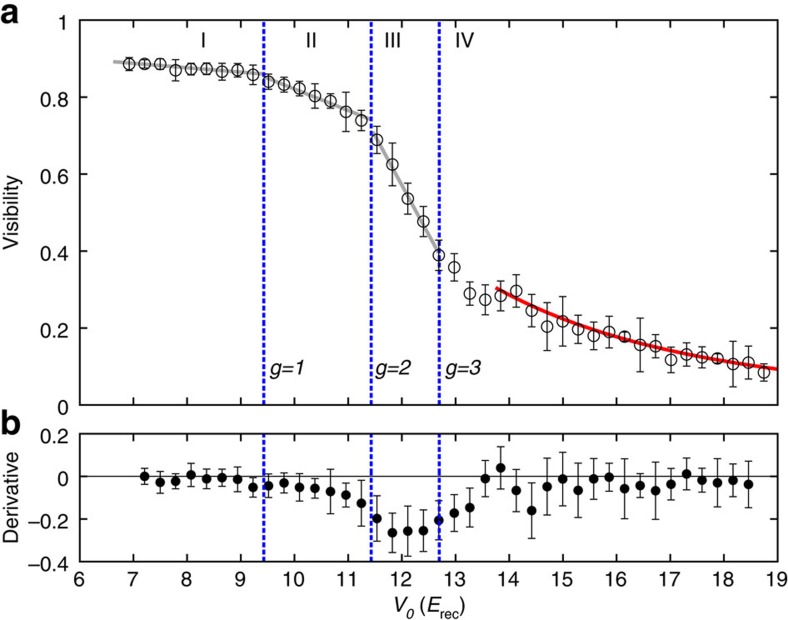
Visibility measurement in the monopartite lattice. (**a**) Visibility of ^87^Rb, plotted as a function of the well depth *V*_0_, for Δ*V*=0 and *V*_z,0_=29 *E*_rec_. Vertical dashed lines: values of *V*_0_/*E*_rec_ corresponding to the tips of the Mott lobes with different filling *g*, as computed through QMC (see Supplementary Note 5). Grey solid lines in regions I–III are a guide to the eyes, whereas the red line in region IV displays a fit to the function *A*(*U*/*zJ*)^*α*^ with *A*=4.0±0.7 and *α*=−1.00±0.06, showing good agreement with the theoretical prediction^18^. The error bars represent the statistical variance of typically 4–5 independent measurements. (**b**) Numerical derivative of the visibility data; vertical lines as in **a**. The error bars are derived from the ones in **a**.

Adjustment of Δ*V*=0 produces the special case of a conventional monopartite square lattice, extensively studied in the literature during the past decade^7^,^18^,^19^,^20^. Experiments in three-dimensional cubic lattices have suggested that the formation of Mott shells within the external trap could be associated with the appearance of kinks in the visibility^18^,^19^, whereas experiments in 2D triangular lattices have rather detected an instantaneous decrease^21^. Arguable attempts were made to interprete small irregularities in the observed visibility in this respect. On the theoretical front, a QMC study of the one-dimensional trapped Bose–Hubbard model^22^ has shown the appearance of kinks in 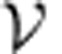 as a function of *U*/*J*. Unfortunately, this study, employing a trap curvature proportional to *J* rather than *V*_0_, appears to have limited relevance for experiments. More realistic QMC simulations of 2D and three-dimensional confined systems have been able to quantitatively describe the momentum distribution^23^ and the experimental visibility^8^,^24^, however, with no indications for distinct features associated with Mott shells. To clarify this long-standing discussion, we have recorded the visibility of Fig. 2 along the Δ*V*=0 trajectory versus *V*_0_ with increased resolution in Fig. 5. Guided by an inhomogeneous mean-field calculation indicating that the local filling *g* is lower than 4, we computed the critical *J*/*U* values for the tips of Mott lobes with *g*=1,2 and 3, making use of the worm algorithm as implemented in the ALPS libraries^25^,^26^,^27^. Superimposed upon the experimental data, we mark in Fig. 5 with (blue) dashed lines the values of *V*_0_/*E*_rec_ corresponding to the values of *J*/*U* at the tip of the Mott lobes obtained by QMC. As *V*_0_ is increased in Fig. 5, four different regimes are crossed. For small values of *V*_0_ (regime I), most of the system is in a superfluid phase. Increasing *V*_0_ yields only little loss of coherence due to increasing depletion, and hence the visibility remains nearly constant. When the first Mott ring with *g*=1 particle per site is formed, the system enters regime II, where the visibility decreases slowly but notably as the *g*=1-Mott shell grows. When the second Mott insulating ring with *g*=2 arises (regime III), a sharp drop of the visibility occurs indicating a significantly increased growth of the Mott insulating part of the system with *V*_0_. Finally, when the third Mott ring with *g*=3 forms or closes in the centre of the trap, only a small superfluid fraction remains in the system, such that the visibility cannot further rapidly decrease with *V*_0_ (regime IV), that is, a quasi-plateau arises in Fig. 5. The red solid line shows that for large *V*_0_ the visibility acquires a (*U*/*J*)^−1^ dependence, in agreement with a result obtained by the first-order perturbation theory in *J*/*U* (ref. 18).

## Discussion

Several conclusions can be drawn from our experimental and theoretical investigations: for monopartite lattices, the visibility comprises characteristic signatures, which can be connected to the position of the tips of the Mott insulator lobes in a *μ*/*U* versus *J*/*U* phase diagram calculated by QMC. Mean-field calculations are insufficient, even when the inhomogeneity due to the trap is taken into account. Deforming the unit cell of a bipartite lattice is a means to efficiently tune a transition from a superfluid to a Mott insulating state. The visibility displays distinct regions with explicitly different slopes, as a function of the detuning between the A and B sublattices. A pronounced loss of coherence occurs at the critical value of the detuning Δ*V*_c_, at which the population of the shallow wells vanish. Our work may shed some light also on the behaviour of condensed-matter systems, where loss of phase coherence occurs due to a structural modification of the lattice. For example, in La_2−*x*_Ba_*x*_CuO_4_ high-*T*_c_ cuprate, superconductivity is weakened at the structural transition from a low-temperature orthorhombic into a low-temperature tetragonal phase^28^. The same occurs for La_2−*x*−*y*_Nd_*y*_Sr_*x*_CuO_4_ (ref. 5). This structural transition corresponds to a buckling of the oxygen octahedra surrounding the copper sites, which changes the nature of the copper–oxygen lattice unit cell^28^. The critical buckling angle *θ*_c_=3.6 deg for the destruction of superconductivity^29^ bears similarities with the critical deformation angle *θ*_c_ (or equivalently Δ*V*_c_) found here (see Supplementary Note 6 for a more detailed discussion). Most of the present theoretical studies of high-*T*_c_ superconductivity concentrate only on the copper lattice. We hope that our results will inspire further investigations of the specific role played by the oxygen lattice, and its importance in preserving A phase coherence

## Methods

### Experimental details

Our experimental procedure begins with the production of a nearly pure Bose–Einstein condensate of typically 5 × 10^4^ rubidium atoms (^87^Rb) in the *F*=2, *m*_F_=2 state confined in a nearly isotropic magnetic trap with about 30 Hz trap frequency. The adjusted values of the lattice depth *V*_0_ are determined with a precision of about 2% by carefully measuring the resonance frequencies with respect to excitations into the third band along the *x*- and *y*-directions. The adjustment of *θ* is achieved with a precision exceeding *π*/300 by an active stabilization with about 10 kHz bandwidth. In a typical experimental run, the lattice potentials *V*(*x*, *y*) and *V*_z_(*z*) are increased to the desired values by an exponential ramp of 160 ms duration. After holding the atoms in the lattice for 20 ms, momentum spectra are obtained by rapidly (<1 μs) extinguishing the lattice and trap potentials, permitting a free expansion of the atomic sample during 30 ms, and subsequently recording an absorption image. The magnetic trap and the finite Gaussian profile of the lattice beams (beam radius=100 μm) give rise to a combined trap potential. For *V*_z,0_=29*E*_rec_ and *V*_0_=18*E*_rec_ this yields trap frequencies of 73 Hz in the *xy*-plane and 65 Hz along the *z*-direction. The observed momentum spectra comprise pronounced Bragg maxima with a visibility depending on the parameters *V*_0_ and Δ*V*. These spectra are analysed by counting the atoms (*n*_d,0_) in a disk with a 5-pixel radius around some higher-order Bragg peak and within a disk of the same radius, but rotated with respect to the origin by 45° (*n*_d,45_). The visibility is obtained as 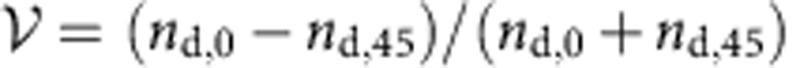 (ref. 18).

### Gutzwiller scheme

The Gutzwiller ansatz approximation used in this work is an extension of the well-known procedure employed for the Bose–Hubbard model in conventional monopartite lattices^16^,^17^ that takes into account the different local energies for the sites of type A and B. The wavefunction is assumed to be a product of single-site wavefunctions |*φ*›=Π_*i*_|*φ*_*i*_›. On each site the ansatz reads


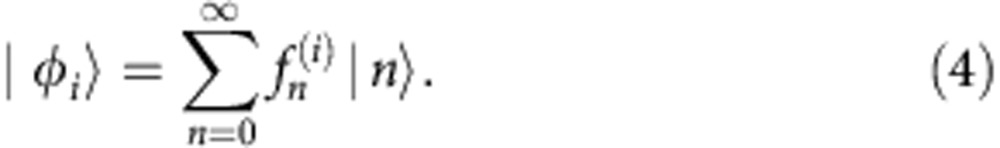


We have included states up to *n*=7 and considered real Gutzwiller coefficients for an extended 69 × 69 lattice, which is allowed because of the U(1) symmetry and the fact that the ground state cannot have nodes, according to Feynman’s no-node theorem.

As shown in Supplementary Note 2, the mean-field Hamiltonian can be written as a sum of site-decoupled local Hamiltonians represented in the local Fock basis, *H*_MF_=∑_*i*_*H*_*i*_. Each local Hamiltonian needs, as an input, the order parameters of the neighbour sites (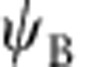 for the local Hamiltonian on sites of type A and vice versa). One can thus use the following iterative procedure to determine the ground state at a given value of *J*/*U* and 
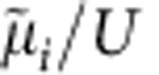
: start with a random guess of the order parameters *ψ*_A,B_, diagonalize the local Hamiltonians *H*_*i*_, take the eigenvectors of the lowest energy state (that is, the Gutzwiller coefficients 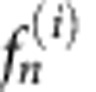), calculate the new order parameters 
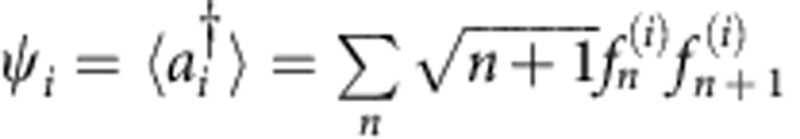
 and repeat the procedure until convergence. In this way, we have obtained Fig. 3 and Supplementary Fig. 6 for the density profiles. By collecting the points where the fraction *n*_B_ of particles on the B sites vanishes, as a function of Δ*V*/*V*_0_, for several values of *V*_0_, we find the white line plotted in Fig. 2.

## Author contributions

M.D.L. and T.C. carried out the calculations; T.K. and M.Ö. carried out the measurements; A.H. and C.M.S. devised the experimental and theoretical parts of the research, respectively. C.M.S. and A.H. wrote the manuscript with input from all authors.

## Additional information

**How to cite this article:** Di Liberto, M. *et al*. Controlling coherence via tuning of the population imbalance in a bipartite optical lattice. *Nat. Commun.* 5:5735 doi: 10.1038/ncomms6735 (2014).

## Supplementary Material

Supplementary Information Supplementary Figures 1-11, Supplementary Notes 1-6 and Supplementary References.
