# Supplementary material for: Controlling coherence via tuning of the population imbalance in a bipartite optical lattice
Source: Nat Commun. 2014 Dec 11;5:5735. doi: 10.1038/ncomms6735 (PMC4284656; doi:10.1038/ncomms6735)
Supplement: Supplementary Information — Supplementary Figures 1-11, Supplementary Notes 1-6 and Supplementary References. [file ncomms6735-s1.pdf]

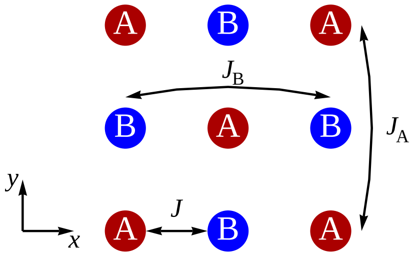

Supplementary Figure 1: Hopping processes included in the tight-binding model.

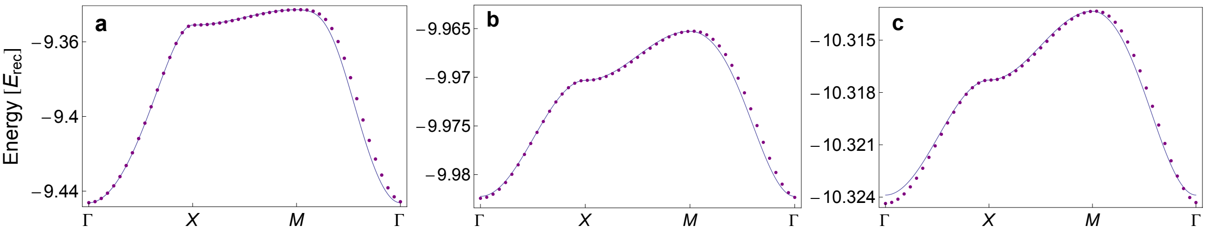

Supplementary Figure 2: Lowest band in the bipartite optical potential. The lowest energy band for $V_0 = 7\ E_{\rm rec}$ and (a) $\theta = 0.502\,\pi$, (b) $\theta = 0.52\,\pi$, and (c) $\theta = 0.53\ \pi$ is plotted versus k, along the high-symmetry lines of the first Brillouin zone. The dots are the results of the exact diagonalization, the solid line is calculated according to the tight-binding Hamiltonian (2) with parameter values determined according to Eq. (4).

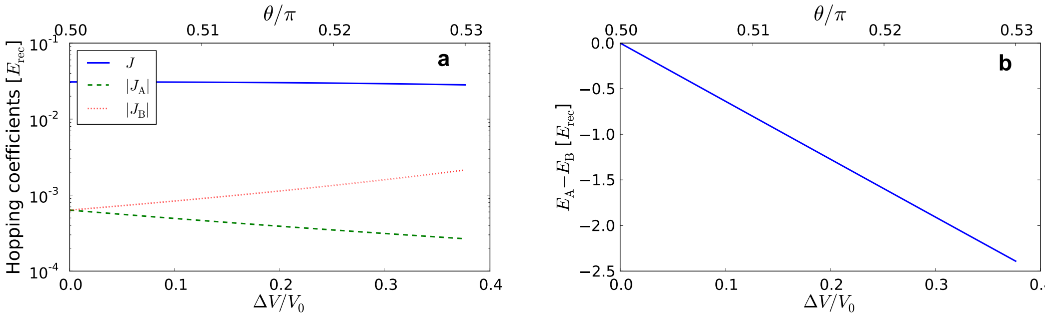

Supplementary Figure 3: Tight-binding parameters. (a) Hopping coefficients and (b) energy difference $E_{\rm A} - E_{\rm B}$ versus ΔV (or equivalently θ) according to Eq. (4) for $V_0 = 8\,E_{\rm rec}$.

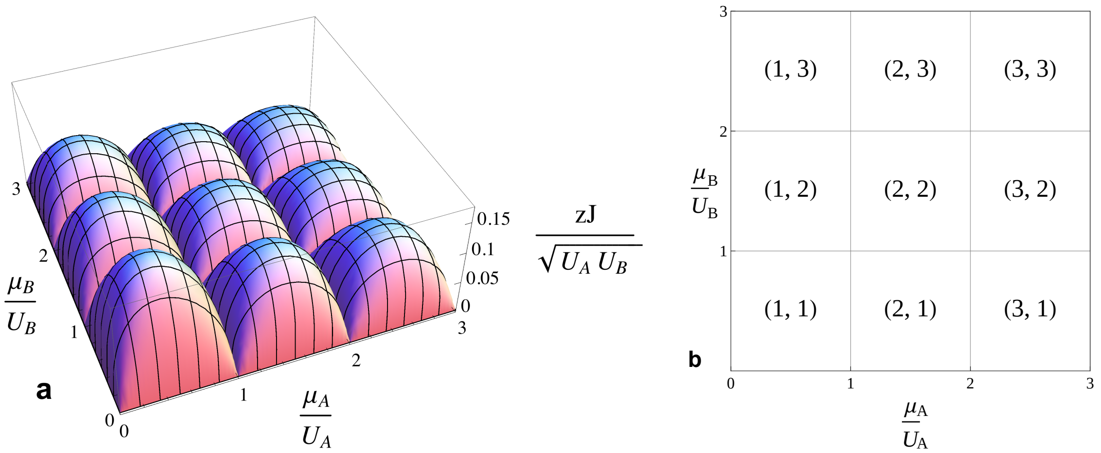

Supplementary Figure 4: Mean-field phase diagram in the bipartite lattice. (a) Phase boundaries: inside each lobe there is a Mott insulator phase with occupations that can differ in the two sublattices according to the chemical potentials, above the lobes the gas is superfluid. (b) Configurations of the occupation numbers $(g_{\rm A}, g_{\rm B})$ inside each lobe.

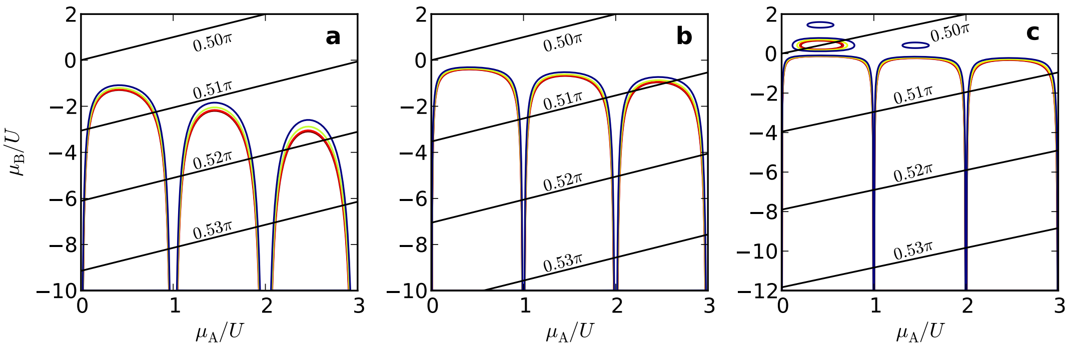

Supplementary Figure 5: Sections through the mean-field phase diagram of the bipartite lattice. For (a) $V_0 = 8E_{\rm rec}$, (b) $V_0 = 10E_{\rm rec}$, and (c) $V_0 = 12E_{\rm rec}$, sections through the phase diagram in Fig. 4 at fixed values of J/U are shown. In each panel, the small change of the Mott lobe boundaries with θ is indicated by contours of different colors; the largest lobe corresponds to the largest value of θ, i.e. the lowest value of J/U (see Supplementary Figure 3a). The diagonal lines are given by Eq. (13), for different values of θ (or equivalently, different values of $\Delta V/V_0$).

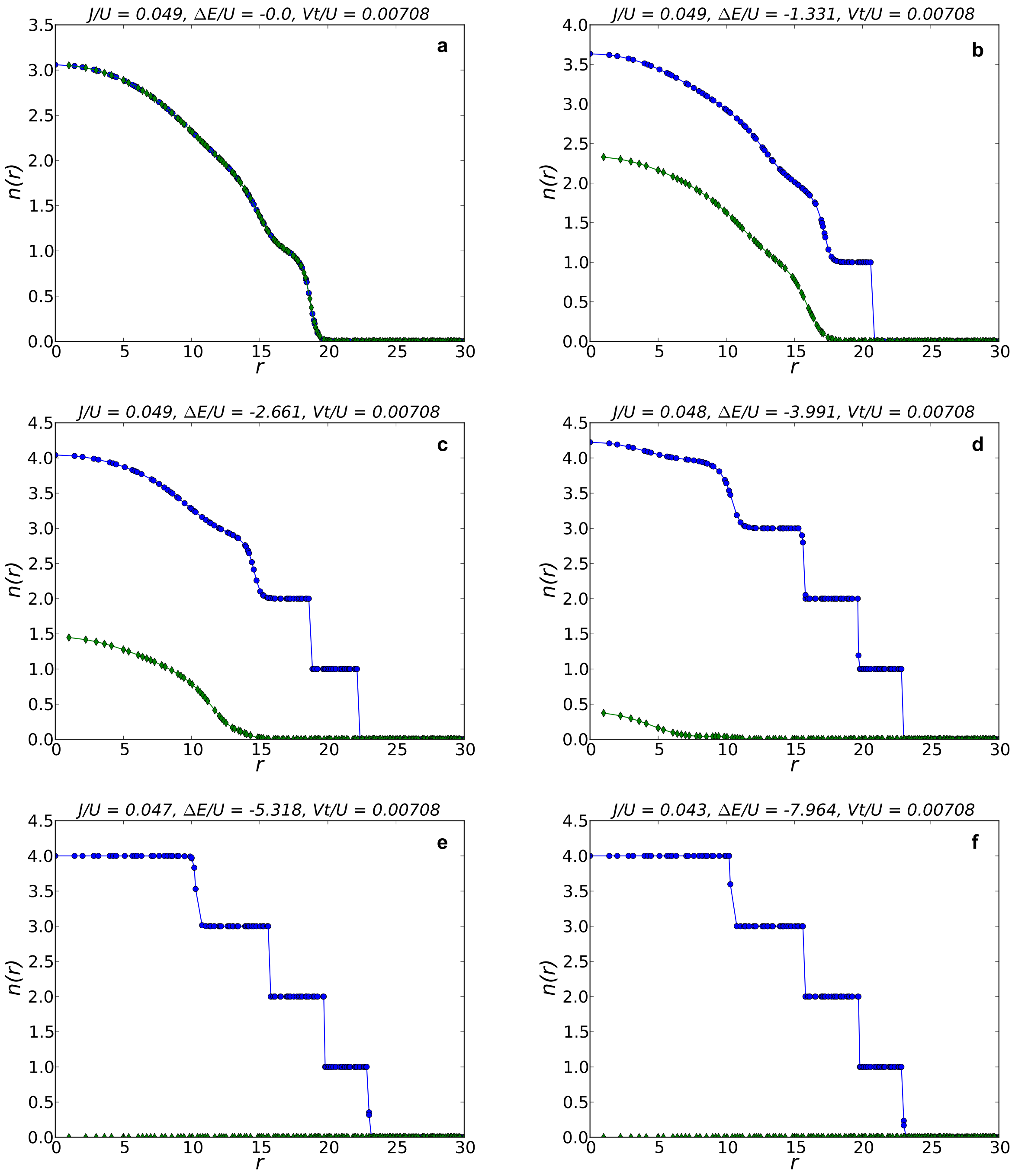

Supplementary Figure 6: Density profiles obtained using the Gutzwiller ansatz for an extended 69×69 lattice in the presence of an harmonic trap for (a) $\theta = 0.5$, (b) $\theta = 0.505$, (c) $\theta = 0.51$,(d) $\theta = 0.515$, (e) $\theta = 0.52$, (f) $\theta = 0.53$ at $V_0 = 10E_{\rm rec}$. Circles (diamonds) denote A (B) sites.

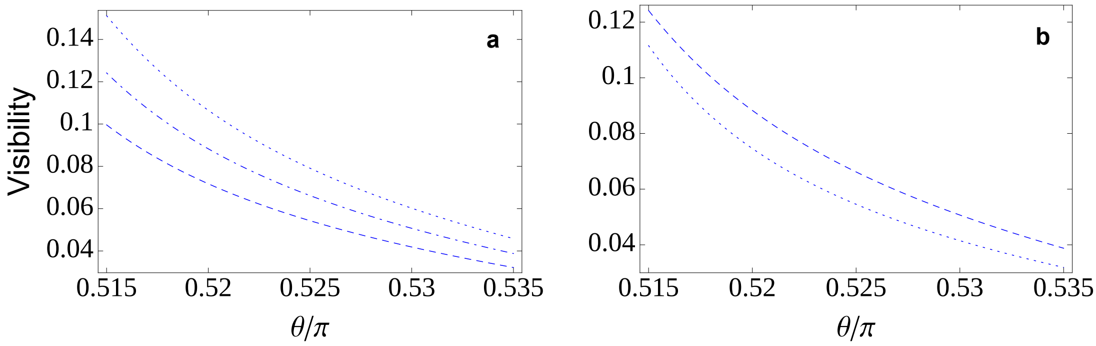

Supplementary Figure 7: Visibility curves calculated within perturbation theory for $V_0 = 10.8\,E_{\rm rec}$. (a) Visibility up to second order for average filling $\bar{g} = 2$ (dashed), $\bar{g} = 2.5$ (dash-dotted), $\bar{g} = 3$ (dotted). (b) Comparison of the visibility curves including contributions up to first order (dotted) and up to second order (dashed) for average filling $\bar{g} = 2.5$.

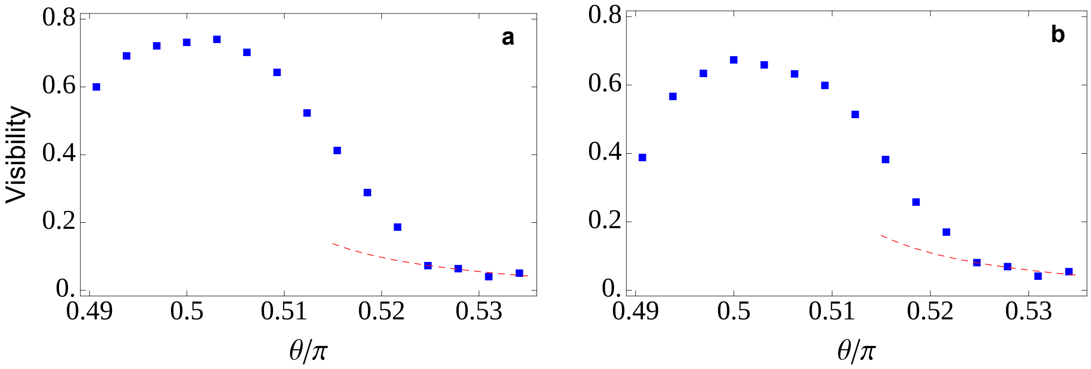

Supplementary Figure 8: Visibility data for (a) $V_0 = 10.8\,E_{\rm rec}$ and (b) $V_0 = 11.44\,E_{\rm rec}$. The red dashed line is obtained by fitting the last four data points with Eq. (21) using the average filling $\bar{g}$ as a fitting parameter. We obtain (a) $\bar{g} = 2.75 \pm 0.23$ and (b) $\bar{g} = 3.77 \pm 0.31$.

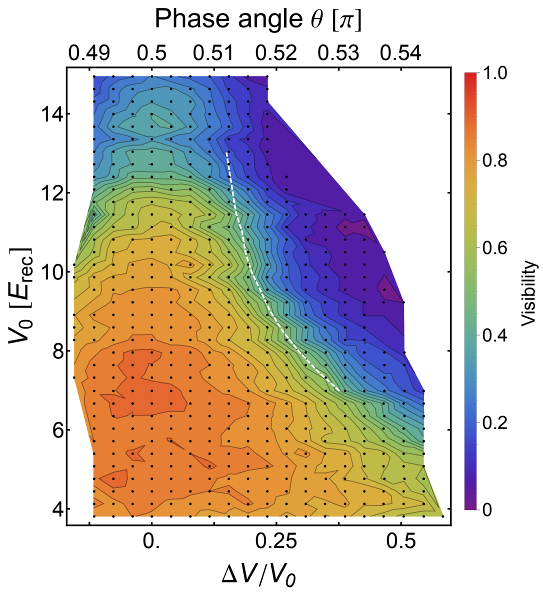

Supplementary Figure 9: Experimental visibility data. The values of $\Delta V/V_0$ and V_0 where measurements were taken are indicated by the black dots. The measured visibility is parametrized by the color scale on the right. The solid light-grey lines show interpolation contours.The white dashed line is a theoretical result for the critical values at which the population of the B sites vanish, obtained without fitting parameters.

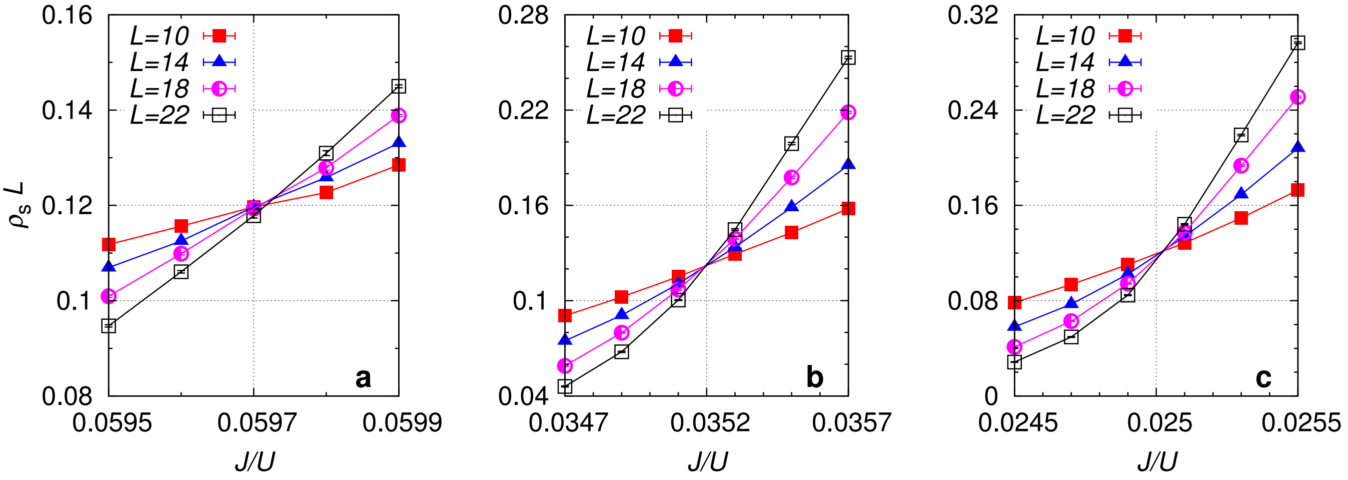

Supplementary Figure 10: Finite-size scaling of the superfluid stiffnes. Values of the product of the superfluid stiffness and the system size $\rho_S L$ are shown as a function of J/U, for different values of the linear size L. The three plots correspond to the tips of the lobes with (a) $g = 1$, (b) $g = 2$ and (c) $g = 3$. Statistical errors are smaller than symbol sizes.

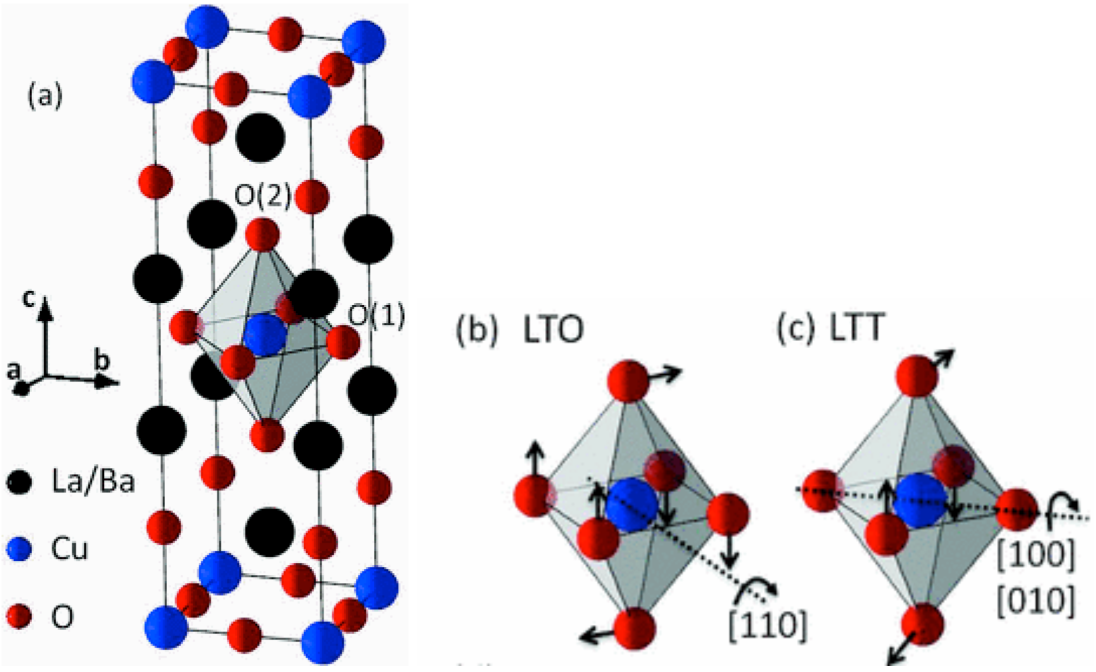

Supplementary Figure 11: (a) Crystal structure of $La_{2-x}Ba_xCuO_4$ or $La_{2-x}Sr_xCuO_4$; (b) buckling of the oxygen octahedra in the LTO and LTT phases. Figure extracted from Ref. [18]

SUPPLEMENTARY NOTE 1. BAND-STRUCTURE AND TIGHT-BINDING MODEL

We employ an optical lattice with two classes of wells (denoted as A and B) arranged as the black and white fields of a chequerboard. The optical potential is

$$V(x,y) = -V_0\left[\cos^2(kx) + \cos^2(ky) + 2\cos\theta\cos(kx)\cos(ky)\right] \tag{1}$$

with the tunable well depth parameter V_0 and the lattice distortion angle θ. Adjustment of θ permits controlled tuning of the well depth difference $\Delta V \equiv 4\,V_0\cos(\theta)$ between A and B sites. In the special case $\theta = \pi/2$ ($\Delta V = 0$) both types of sites are equivalent and hence a monopartite lattice arises, while in general the lattice is bipartite. Using the potential of Eq. (1), we have numerically solved the Schrödinger equation for the single particle problem to obtain the exact band structure, including 14 bands in the plane-wave matrix representation of the Hamiltonian [1].

The single-particle problem is reformulated in terms of a tight-binding model Hamiltonian,

$$H = -J\sum_{\langle i,j\rangle}(a_i^\dagger a_j + \text{h.c.}) - J_\text{A}\sum_{\langle i,j\rangle_\text{A}}(a_i^\dagger a_j + \text{h.c.}) - J_\text{B}\sum_{\langle i,j\rangle_\text{B}}(a_i^\dagger a_j + \text{h.c.}) + E_\text{A}\sum_{i\in\text{A}} n_i + E_\text{B}\sum_{i\in\text{B}} n_i\,, \tag{2}$$

where J is the hopping between neighboring sites of different sublattices, J_A (J_B) is the hopping coefficient between neighboring sites of sublattice A (B) (see Supplementary Figure 1), and E_A (E_B) is the on-site energy of sites belonging to sublattice A (B). We neglect the $A \to A$ hopping (henceforth indicated as J'_A) along the diagonal lines of the lattice (same for $B \to B$), because for the monopartite lattice ($\theta = \pi/2$ or $\Delta V = 0$) these hopping coefficients are exactly zero as a consequence of the symmetry of the Wannier functions. For sufficiently small deviations from $\theta = \pi/2$, we expect that these coefficients are still negligible compared to J_A or J_B; this assumption is supported by the full band structure calculation. For $\theta \gtrsim 0.53\,\pi$, this assumption becomes less reliable (see Supplementary Figure 2).

By diagonalizing the Hamiltonian in Eq. (2) in momentum space and taking the lattice constant to unity, an analytic expression for the corresponding band structure (depending on the parameters $E_\text{A}, E_\text{B}, J, J_\text{A}, J_\text{B}$) can be derived,

$$H(\mathbf{k}) = \begin{pmatrix} E_\text{A} - 4J_\text{A}\cos(2k_x)\cos(2k_y) & -4J\cos(k_x)\cos(k_y) \\ -4J\cos(k_x)\cos(k_y) & E_\text{B} - 4J_\text{B}\cos(2k_x)\cos(2k_y) \end{pmatrix}. \tag{3}$$

When θ is tuned away from zero a gap opens, splitting the lowest band. We denote the two resulting bands by "1" and "2", with the corresponding energies $E_1(k_x,k_y)$ and $E_2(k_x,k_y)$. It is straightforward to verify that

$$\begin{aligned} E_\text{A} &= E_1(\pi/2,\pi/4)\,, \\ E_\text{B} &= E_2(\pi/2,\pi/4)\,, \\ J &= \frac{1}{4}\sqrt{(E_1(\pi/4,\pi/4) - E_2(\pi/4,\pi/4))^2 - (E_\text{A} - E_\text{B})^2}\,, \\ J_\text{A} &= \frac{1}{8}(E_1(\pi/2,0) - E_1(\pi/2,\pi/2))\,, \\ J_\text{B} &= \frac{1}{8}(E_2(\pi/2,0) - E_2(\pi/2,\pi/2))\,. \end{aligned} \tag{4}$$

In order to determine the parameters of the model Hamiltonian (2), instead of calculating Wannier functions, we use these equations to adjust the tight-binding bands to the exact band structure calculation, finding reasonable agreement up to $\theta = 0.53\,\pi$, as shown in Supplementary Figure 2. The resulting values of the hopping coefficients and the energy difference $E_\text{A} - E_\text{B}$ are plotted in Supplementary Figure 3. Since $|J_\text{A}|$ and $|J_\text{B}|$ are nearly two orders of magnitude smaller than J, we will neglect them in what follows, as long as $J \neq 0$.

SUPPLEMENTARY NOTE 2. MEAN-FIELD PHASE DIAGRAM OF THE BIPARTITE LATTICE MODEL

In this section, we summarize the calculation of the mean-field phase diagram of the bosonic model

$$H = -J\sum_{\langle i,j\rangle}(a_i^\dagger a_j + \text{h.c.}) - \mu_\text{A}\sum_{i\in A} n_i - \mu_\text{B}\sum_{i\in B} n_i + \frac{U_\text{A}}{2}\sum_{i\in A} n_i(n_i-1) + \frac{U_\text{B}}{2}\sum_{i\in B} n_i(n_i-1). \tag{5}$$

We restrict ourselves to nearest-neighbor hopping coefficients, which are the only relevant ones, as shown in Supplementary Figure 3. The Hamiltonian in Eq. (5) describes a bipartite lattice, in which one allows for different densities in the two sublattices. A similar problem has been discussed also in Ref. [2]. Extending a standard approach [3, 4], we apply a mean-field decoupling of the hopping term [first term in Eq. (5)],

$$a_{i(\text{A})} \to \psi_\text{A} + \delta a_{i(\text{A})}\,, \qquad a_{i(\text{B})} \to \psi_\text{B} + \delta a_{i(\text{B})} \tag{6}$$

with the order parameters $\psi_{\mathrm{A,B}} \equiv \langle a_{i(\mathrm{A,B})}\rangle$ and the fluctuations $\delta a_{i(\mathrm{A,B})}$. Neglecting the second order fluctuations of the fields, one finds

$$H_J \simeq -4J\sum_{i\in \mathrm{A}}\left(\psi_{\mathrm{B}}a_i+\psi_{\mathrm{B}}^*a_i^\dagger\right)-4J\sum_{i\in \mathrm{B}}\left(\psi_{\mathrm{A}}a_i+\psi_{\mathrm{A}}^*a_i^\dagger\right)+4N_{\mathrm{A}}J(\psi_{\mathrm{A}}^*\psi_{\mathrm{B}}+\psi_{\mathrm{A}}\psi_{\mathrm{B}}^*)\equiv H_{J0}+4N_{\mathrm{A}}J(\psi_{\mathrm{A}}^*\psi_{\mathrm{B}}+\psi_{\mathrm{A}}\psi_{\mathrm{B}}^*)\,, \quad (7)$$

where N_{A} denotes the number of sites in the sublattice A. We use H_{J0} as a perturbation to the interaction part of the Hamiltonian (5), and neglect for the moment the irrelevant constant shift given by the last term in Eq. (7). Since H_{J0} is local, the total Hamiltonian contains only local terms and we can apply perturbation theory in each unit cell. The unperturbed Hamiltonian $H(J=0)$ is diagonal with respect to the number operators and, hence, the eigenstates of $H(J=0)$ in each unit cell are $|n_{\mathrm{A}}, n_{\mathrm{B}}\rangle$, where n_{A} and n_{B} are the occupation numbers of the sites A and B, respectively. The energy per unit cell is given by

$$E(n_{\mathrm{A}},n_{\mathrm{B}})=\frac{U_{\mathrm{A}}}{2}n_{\mathrm{A}}(n_{\mathrm{A}}-1)+\frac{U_{\mathrm{B}}}{2}n_{\mathrm{B}}(n_{\mathrm{B}}-1)-\mu_{\mathrm{A}}n_{\mathrm{A}}-\mu_{\mathrm{B}}n_{\mathrm{B}}\,. \quad (8)$$

The ground state corresponds to occupations g_{A} and g_{B} determined by the relations $U_\nu(g_\nu-1)<\mu_\nu<U_\nu g_\nu$, with $\nu = A, B$. The first order contribution of the perturbation H_{J0} vanishes because H_{J0} does not conserve the number of particles, whereas the second order is found to be

$$\begin{aligned} E^{(2)} &= \sum_{(n_{\mathrm{A}},n_{\mathrm{B}})\neq(g_{\mathrm{A}},g_{\mathrm{B}})}\frac{|\langle g_{\mathrm{A}},g_{\mathrm{B}}|H_{J0}|n_{\mathrm{A}},n_{\mathrm{B}}\rangle|^2}{E(g_{\mathrm{A}},g_{\mathrm{B}})-E(n_{\mathrm{A}},n_{\mathrm{B}})}\\ &=(4J)^2\left[\frac{|\psi_{\mathrm{B}}|^2g_{\mathrm{A}}}{U_{\mathrm{A}}(g_{\mathrm{A}}-1)-\mu_{\mathrm{A}}}+\frac{|\psi_{\mathrm{B}}|^2(g_{\mathrm{A}}+1)}{\mu_{\mathrm{A}}-U_{\mathrm{A}}g_{\mathrm{A}}}+\frac{|\psi_{\mathrm{A}}|^2g_{\mathrm{B}}}{U_{\mathrm{B}}(g_{\mathrm{B}}-1)-\mu_{\mathrm{B}}}+\frac{|\psi_{\mathrm{A}}|^2(g_{\mathrm{B}}+1)}{\mu_{\mathrm{B}}-U_{\mathrm{B}}g_{\mathrm{B}}}\right]. \end{aligned} \quad (9)$$

Including the previously ignored constant shift and using the fact that at zero temperature the calculated energy is the same as the Helmholtz free energy F, we can write

$$F[\psi_{\mathrm{A}},\psi_{\mathrm{B}}]=F^{(0)}+\sum_{\mu,\nu=A,B}\psi_\mu^*M_{\mu\nu}\psi_\nu \quad (10)$$

with

$$F^{(0)}=\frac{U_{\mathrm{A}}}{2}g_{\mathrm{A}}(g_{\mathrm{A}}-1)+\frac{U_{\mathrm{B}}}{2}g_{\mathrm{B}}(g_{\mathrm{B}}-1)-\mu_{\mathrm{A}}g_{\mathrm{A}}-\mu_{\mathrm{B}}g_{\mathrm{B}} \quad (11)$$

and

$$\mathbf{M}=\begin{pmatrix}\left(\frac{g_{\mathrm{B}}}{U_{\mathrm{B}}(g_{\mathrm{B}}-1)-\mu_{\mathrm{B}}}+\frac{g_{\mathrm{B}}+1}{\mu_{\mathrm{B}}-U_{\mathrm{B}}g_{\mathrm{B}}}\right)J^2z^2 & zJ\\ zJ & \left(\frac{g_{\mathrm{A}}}{U_{\mathrm{A}}(g_{\mathrm{A}}-1)-\mu_{\mathrm{A}}}+\frac{g_{\mathrm{A}}+1}{\mu_{\mathrm{A}}-U_{\mathrm{A}}g_{\mathrm{A}}}\right)J^2z^2\end{pmatrix}. \quad (12)$$

Here, $z=2d$ is the coordination number of the lattice; in our case $d=2$ and $z=4$. According to the (generalized) Landau criterion for continuous phase transitions, the phase boundaries are given by the condition $\mathrm{Det}[\mathbf{M}]=0$. In the phase diagram shown in Supplementary Figure 4(a), one observes a series of lobes corresponding to Mott-insulator phases with occupation numbers that can vary in the two sublattices according to the value of the chemical potentials (see also Supplementary Figure 4(b), where the $(g_{\mathrm{A}}, g_{\mathrm{B}})$ filling of the Mott lobes is explicitly given). Outside the lobes the system is superfluid.

SUPPLEMENTARY NOTE 3. EFFECT OF THE TRAP

We now discuss the effect of the additional harmonic trap potential. We set $U_{\mathrm{A}}=U_{\mathrm{B}}=U$, which is a very good approximation for $\theta \lesssim 0.53\,\pi$. In Supplementary Figure 5, horizontal sections through the mean-field phase diagram are plotted for fixed values of V_0. The lobes for $\mu_{\mathrm{B}}<0$ correspond to Mott phases with occupations $(g_{\mathrm{A}}, g_{\mathrm{B}}) = (g, 0)$, with g integer. For different values of θ, we also plot the lines $\mathcal{L}(\theta)$ given by

$$\mu_{\mathrm{B}}-\mu_{\mathrm{A}}=\Delta\mu(\theta)\,, \quad (13)$$

where $\Delta\mu(\theta)=E_{\mathrm{A}}-E_{\mathrm{B}}$ is the difference of the local energies E_{A} and E_{B} determined through Eq. (4). According to the local density approximation, one can define a local chemical potential with a maximal value in the center of the trap fixed by the total particle number, which decreases towards the edge of the trap. Hence, the phases encountered locally along a radial path pointing outwards from the trap center are given by the homogeneous phase diagram, when following the lines $\mathcal{L}(\theta)$ towards decreasing values of μ_{A}/U. The lines $\mathcal{L}(\theta)$ shift to large, negative values of μ_{B}/U as θ increases. As discussed in the main text, this means that the population of the B sites decreases and eventually

vanishes. Hence, the density profile evolves into a wedding cake structure where only the A sites are populated, i.e., most atoms contribute to pure A-site Mott shells $(g,0)$ separated by narrow superfluid films, also with negligible B population (see Supplementary Figure 6 for an example of density profiles calculated with the Gutzwiller ansatz). The plot also shows that for increasing V_0 the Mott lobes $(g,0)$ cover an increasing area in the phase diagram, while at the same time the lines $\mathcal{L}(\theta)$ shift towards lower values of $\mu_{\rm B}/U$. This explains why the value of $\Delta V_{\rm c}$, at which one observes a sudden loss of the visibility, reduces when V_0 is increased.

SUPPLEMENTARY NOTE 4. PERTURBATIVE RESULTS FOR THE VISIBILITY IN THE ASYMPTOTIC LIMIT.

The regime where the imbalance between A and B sites is large can be studied using perturbation theory up to second order [5] when the filling is chosen to be integer in the homogeneous case. In the limit where the hopping term is neglected (which is also the mean-field ground state), the ground state is given by a perfect Mott insulator of the form $(g_{\rm A}, g_{\rm B}) = (g, 0)$

$$|MI\rangle = \prod_{i\in A} |g\rangle_i \prod_{j\in B} |0\rangle_j \,. \tag{14}$$

Let us start from the first order term. The only non-vanishing terms are the ones for which a particle is removed from a site A and moved to one of the nearest-neighbor B sites. The energy difference is $\Delta = U(g-1) + \Delta\mu$ and the first order correction has thus the form

$$-\frac{J}{\Delta}\sum_{\langle i,j\rangle} a_i^\dagger a_j |MI\rangle \,. \tag{15}$$

The quadratic correction is such that a particle is removed from an A site, moved to a nearest-neighbor B site and from there it is transferred again to an A site which is different from the original one. The final A site can be a nearest-neighbor A site or a next-nearest-neighbor one. The correction becomes

$$-\frac{2J^2}{U\Delta}\sum_{\langle i,j\rangle_{\rm A}} a_i^\dagger a_j |MI\rangle - \frac{J^2}{U\Delta}\sum_{\langle\langle i,j\rangle\rangle_{\rm A}} a_i^\dagger a_j |MI\rangle \,. \tag{16}$$

The ground state is therefore

$$|\psi_G\rangle = \left(1 - \frac{J^2}{2\Delta^2}\right)|MI\rangle - \frac{J}{\Delta}\sum_{\langle i,j\rangle} a_i^\dagger a_j |MI\rangle - \frac{2J^2}{U\Delta}\sum_{\langle i,j\rangle_{\rm A}} a_i^\dagger a_j |MI\rangle - \frac{J^2}{U\Delta}\sum_{\langle\langle i,j\rangle\rangle_{\rm A}} a_i^\dagger a_j |MI\rangle \,, \tag{17}$$

where the first term is simply the unperturbed term with a wave function renormalilzation.

We can now calculate the momentum distribution

$$S(\mathbf{k}) = \frac{1}{N_s}\sum_{i,j} e^{i\mathbf{k}\cdot(\mathbf{r}_i - \mathbf{r}_j)} \langle a_i^\dagger a_j\rangle \,, \tag{18}$$

where N_s is the number of unit cells in the system. The visibility $\mathcal{V}$ is calculated at momenta $k_{\rm max} = (0,0)$ and $k_{\rm min} = (\sqrt{2}\pi, \sqrt{2}\pi)$. Therefore,

$$S_{\rm max} = \left(1 - \frac{J^2}{\Delta^2}\right) g - 8g(g+1)\frac{J}{\Delta}\left(\frac{3J}{U} + 1\right) \,, \tag{19}$$

$$S_{\rm min} = \left(1 - \frac{J^2}{\Delta^2}\right) g - 4g(g+1)\left[\frac{J}{\Delta}(r_1+1) + \frac{2J^2}{U\Delta}(2r_1 + r_2)\right] \,, \tag{20}$$

where $r_1 \equiv \cos(\sqrt{2}\pi) \approx -0.266$ and $r_2 \equiv \cos(\sqrt{8}\pi) \approx -0.858$, and we eventually find

$$\begin{aligned} \mathcal{V} &= (S_{\rm max} - S_{\rm min})/(S_{\rm max} + S_{\rm min}) \\ &= -2(g+1)(1-r_1)\frac{J}{\Delta} + (g+1)(2r_1 + r_2 - 3)\frac{4J^2}{\Delta U} - 4(g+1)^2(r_1+3)(1-r_1)\frac{J^2}{\Delta^2} \,. \end{aligned} \tag{21}$$

The visibility obtained in Eq. (21) is of the order 10^{-1} in the highly imbalanced regime for filling between 2 and 3 (see Supplementary Figure 7(a)). The second order processes contribute significantly, as can be observed in Supplementary Figure 7(b). In the theory just discussed, we did not include the contributions given by the bare next-nearest-neighbor hopping processes ($J_{\rm A}$), despite the fact that the ground state (17) effectively includes this type of hopping contributions through virtual transitions. The reason is that the effective hopping processes contribute more substantially to the visibility than the bare ones (not displayed here).

In Supplementary Figure 8, the experimental data for the visibility (extracted from Supplementary Figure 9) are plotted for $V_0 = 10.8\,E_{\rm rec}$ and $V_0 = 11.44\,E_{\rm rec}$. The behavior of the visibility at large imbalance, where the system is deeply in a Mott insulator phase, can be described by Eq. (21), where the average filling $\bar{g}$ has been used as a fitting parameter.

SUPPLEMENTARY NOTE 5. QUANTUM MONTE CARLO RESULTS IN 2D

Here, we describe the QMC procedure used to obtain the results for the critical values of the interaction at the tip of the $g = 1, 2, 3$ lobes in the phase diagram of the homogeneous Bose-Hubbard model for the monopartite lattice. We make use of the worm algorithm, as implemented in the ALPS libraries [6, 7]. By measuring the superfluid stiffness, we are able to distinguish between the two phases of the homogeneous system on a square lattice. We use a finite-size scaling to determine the position of the critical point, keeping the product of the temperature T and the linear size L of the lattice constant [8]: $T \times L = 0.1\,U$. The comparison with a precise QMC calculation at zero temperature for filling $g = 1$ [9] allows us to conclude that the choice of temperature is adequate to describe the zero-temperature system.

We study the system for different values of the ratio J/U, while keeping the chemical potential constant and equal to $\mu/U = 0.371, 1.427, 2.448$, for $g = 1, 2, 3$, respectively. The choice of μ for $g = 1$ is comparable with the results in Ref. [9], while the choices of μ for $g = 2$ and $g = 3$ are taken from Ref. [10]. We set the maximum on-site occupation number to be $g + 2$ (and $g + 3$ for $g = 1$), thus allowing for more processes than just particle-hole excitations.

Our goal is to evaluate the lobe positions using a more reliable method than the usual mean-field approach [4]. We stress that a higher precision in the determination of the critical points, that could be obtained by lowering the temperature, increasing the system size and using a finer scan of the area around the lobe tip, is beyond the scope of this work, as it would not be relevant in the comparison with experimental results. For $g = 1, 2, 3$, we find the following values of $(J/U)_c$: 0.0597 ± 0.0001, 0.0352 ± 0.0001, 0.0250 ± 0.0001, where the errors are due to the use of a finite grid for J/U. These values are in agreement with a high-precision $T = 0$ result for $g = 1$ [9], and with estimates given in Ref. [10], based on the use of the effective potential method and Kato's perturbation theory (we observe that the latter values of $(J/U)_c$ are systematically smaller than the ones we find). In Supplementary Figure 10, we show the finite-size scaling, as done in Ref. [8].

SUPPLEMENTARY NOTE 6. ANALOGIES TO HIGH-T_c SUPERCONDUCTORS

Our work may shed some light also on the behavior of similar condensed-matter systems, where loss of phase coherence occurs due to a structural modification of the lattice. One possible example are high-T_c cuprates. Although the phenomenon of superconductivity occurs due to paired electrons, and here we are studying bosons, our system could bear some similarities with the cuprates, if one considers the scenario of pre-formed Cooper pairs at a higher temperature scale, as suggested by several theoretical and experimental works [11–14]. In this case, the onset of superconductivity at T_c would correspond simply to phase coherence of the pre-formed "bosons".

The first discovered high-T_c cuprate, $La_{2-x}Ba_xCuO_4$ (see Supplementary Figure 11) was found to exhibit a dip in the critical temperature at the doping value $x = 1/8$. Later, the same phenomenon was shown to occur for $La_{2-x}Sr_xCuO_4$ when La was partially substituted by some rare earth elements, like Eu or Nd [15]. This feature was long known as the 1/8 mystery, but further investigations of the materials have shown that it is connected to a structural transition from a low-temperature orthorhombic (LTO) into a low-temperature tetragonal (LTT) phase [16], see also Supplementary Figure 11. This structural transition corresponds to a buckling of the oxygen octahedra surrounding the copper sites, which changes the nature of the copper-oxygen lattice unit cell [16]. By increasing the concentration y of Nd in $La_{2-x-y}Nd_ySr_xCuO_4$, superconductivity is eventually destroyed. The onset for the disappearance of superconductivity depends also on the Sr doping x, but actually there is a universal critical angle $\theta_c = 3.6\,\text{deg}$ for the buckling of the oxygen octahedra, after which superconductivity cannot survive [17].

Until now, most of the theoretical studies of high-T_c cuprates have concentrated on the 2D square copper lattice, but it is well known that the actual superconducting plane is composed of copper and oxygen forming a Lieb lattice, and that the dopants sit on the oxygen (see Supplementary Figure 11). The role of the LTO/LTT structural transition is mostly to shift two of the four in-plane oxygen atoms, which were slightly out of the plane, back into it. Although essentially more complicated than the problem studied here, the critical buckling angle $\theta_c = 3.6\,\text{deg}$ for the destruction of superconductivity [17] bears similarities with the critical deformation angle θ_c (or equivalently ΔV_c) that we found in this work. We hope that our results will foster further investigations of the specific role played by the oxygen lattice in high-T_c superconductors, and its importance in preserving phase coherence.

SUPPLEMENTARY REFERENCES

[1] Paul, S. & Tiesinga, E. Formation and decay of Bose-Einstein condensates in an excited band of a double-well optical lattice. *Phys. Rev. A* **88**, 033615 (2013).

[2] Chen, B., Kou, S., Zhang, Y., & Chen, S. Quantum phases of the Bose-Hubbard model in optical superlattices. *Phys. Rev. A* **81**, 053608 (2010).

[3] Sheshadri, K., Krishnamurthy, R., Pandit R., & Ramakrishnan, T. V. Superfluid and insulating phases in an interacting-boson model - mean-field theory and the RPA. *Europhys. Lett.* **22**, 257-263 (1993).

[4] van Oosten, D., van der Straten, P. & Stoof, H. T. C. Quantum phases in an optical lattice. *Phys. Rev. A* **63**, 053601 (2001).

[5] J.J. Sakurai, *Modern Quantum Mechanics*, Addison Wesley (2009).

[6] Albuquerque, A. F. *et al.* (ALPS collaboration) The ALPS project release 1.3: Open-source software for strongly correlated systems. *J. of Magn. and Magn. Materials* **310**, 1187 (2007).

[7] Bauer, B. *et al.* (ALPS collaboration) The ALPS project release 2.0: open source software for strongly correlated systems. *J. Stat. Mech.* P05001 (2011).

[8] Šmakov, J. & Sørensen, E. Universal scaling of the conductivity at the superfluid-insulator phase transition. *Phys. Rev. Lett.* **95**, 180603 (2005).

[9] Capogrosso-Sansone, B., Söyler, S. G., Prokof'ev, N. & Svistunov, B. Monte Carlo study of the two-dimensional Bose-Hubbard model. *Phys. Rev. A* **77**, 015602 (2008).

[10] Teichmann, N., Hinrichs, D., Holthaus, M. & Eckardt, A. Bose-Hubbard phase diagram with arbitrary integer filling. *Phys. Rev. B* **79**, 100503(R) (2009).

[11] Pasupathy, A. N. *et al.* Electronic Origin of the Inhomogeneous Pairing Interaction in the High-Tc Superconductor $Bi_2Sr_2CaCu_2O_{8+\delta}$. *Science* **11**, 1154700 (2008).

[12] Deutscher, G. AndreevSaint-James reflections: A probe of cuprate superconductors. *Rev. Mod. Phys.* **77**, 109 (2005)

[13] Emery, V. J. & Kivelson, S. A. Importance of phase fluctuations in superconductors with small superfluid density. *Nature* **374**, 434 (1995).

[14] Randeria, M.,Trivedi, N., Moreo, A., & Scalettar, Richard T. Pairing and spin gap in the normal state of short coherence length superconductors. *Phys. Rev. Lett.* **69**, 13 (2001), Erratum Phys. Rev. Lett. **72**, 3292 (1994).

[15] Tranquada, J. M. Spins, stripes, and superconductivity in hole-doped cuprates. *AIP Conf. Proc.* **1550**, 114 (2013).

[16] Axe, J. D. *et al.* Structural phase transformation and superconductivity in $La_{2-x}Ba_xCuO_4$. *Phys. Rev. Lett.* **62**, 2751 (1989).

[17] Buchner, B. *et al.* Critical Buckling for the Disappearance of Superconductivity in Rare-Earth-Doped $La_{2-x}Sr_xCuO_4$. *Phys. Rev. Lett.* **73**, 1841 (1994).

[18] Fabbris, G., Hücker, M., Gu, G. D., Tranquada, J. M., & Haskel, D. Local structure, stripe pinning, and superconductivity in $La_{1.875}Ba_{0.125}CuO_4$ at high pressure. *Phys. Rev. B* **88**, 060507(R) (2013).
